# Influence of Dentin Hybridization on the Chromatic Alteration of Teeth caused by Endodontic Sealers

**DOI:** 10.1590/0103-6440202405878

**Published:** 2024-12-06

**Authors:** Lucas S Chaves, Daniel A Decurcio, Ana Paula R Magalhães, Gustavo S Chaves, Lucas RA Estrela, Cyntia RA Estrela, Carlos Estrela

**Affiliations:** 1 School of Dentistry, Federal University of Goiás, Goiânia, GO, Brazil.; 2 School of Dentistry, Alfredo Nasser University Center, Aparecida de Goiânia, Goiás, Brazil.; 3 Department of Preventive and Restorative, Dentistry, School of Dentistry, São Paulo State University(UNESP), SP, Brazil;; 4 School of Dentistry, Evangelical University of Goiás, GO, Brazil.

**Keywords:** Dentin adhesives, endodontics, root canal filling, sealer, tooth color

## Abstract

This study evaluated the influence of dentin hybridization on coronary chromatic alteration of endodontically treated bovine teeth using different endodontic sealers and Portland cement. The 200 central incisors were endodontically treated using different materials (Sealapex, Bio-C Sealer, AH-Plus, Endofill, and Portland cement) and distributed according to the presence or absence of dentin hybridization. The teeth underwent colorimetric analysis using the Easyshade® spectrophotometer at four different times: determining the color of the dental substrate before root canal filling (RCF), 7 days, 60 days, and 180 days after RCF. The data were evaluated for normality and homogeneity by the Shapiro-Wilk and Levene tests. A two-way analysis of variance was performed, with Sidak's post-test for multiple comparisons. The significance level was 5%. Dentin hybridization influenced the coronary chromatic alteration of Sealapex® after 7 days and in Bio-C Sealer® and AH Plus® after 60 days. The AH Plus after 7 days obtained the highest ΔE* when hybridized, and the Endofill® obtained the highest ΔE* when not hybridized. The AH Plus after 60 days got the highest ΔE* when hybridized and non-hybridized. The Sealapex® after 180 days obtained the highest ΔE* when hybridized, and the AH Plus obtained the highest ΔE* when not hybridized. The studied root canal sealers behaved differently regarding coronary chromatic alteration, making it difficult to predict a particular behavior. All endodontic sealers caused clinically noticeable chromatic changes after 7 days, 60 days, and 180 days, regardless of hybridization.

## Resumo

Este estudo avaliou a influência da hibridização dentinária na alteração cromática coronária de dentes bovinos tratados endodonticamente, usando diferentes cimentos endodônticos e o cimento Portland. Duzentos incisivos centrais bovinos foram tratados endodonticamente com diferentes materiais (Sealapex®, Bio-C sealer®, AH-Plus®, Endofill®, e o cimento Portland), e distribuídos em 2 grupos, com e sem a hibridação dentinária. Os dentes foram submetidos à análise colorimétrica usando o espectrofotômetro Easyshade® em quatro momentos diferentes: determinação da cor do substrato dental antes da obturação do canal radicular, e posterior aos 7 dias, 60 dias e 180 dias. Os dados foram avaliados quanto à normalidade e homogeneidade pelos testes Shapiro-Wilk e Levene. Foi realizada análise de variância de duas vias, com pós-teste de Sidak para comparações múltiplas. O nível de significância foi de 5%. A hibridação dentinária influenciou a alteração cromática coronária do Sealapex® após 7 dias e do Bio-C Sealer® e AH Plus® após 60 dias. O AH Plus® após 7 dias obteve o maior ΔE* quando hibridizado, e o Endofill® obteve o maior ΔE* quando não hibridizado. O AH Plus após 60 dias obteve o maior ΔE* quando hibridizado e não hibridizado. O Sealapex® após 180 dias obteve o maior ΔE* quando hibridizado, e o AH Plus® obteve o maior variação de cor quando não hibridizado. Os cimentos endodônticos estudados se comportaram de forma diferente em relação à alteração cromática coronária, sendo difícil prever um determinado comportamento. Todos os cimentos endodônticos causaram alterações cromáticas clinicamente perceptíveis após 7 dias, 60 dias e 180 dias, independentemente da hibridação dentinária.

## Introduction

Root canal filling materials (RCFM) are important sealing agents which associated with gutta-percha points and are responsible for eliminating empty spaces in the pulp cavity and can harbor microorganisms. A variety of RCFM and techniques have been proposed over the years, whose main objective was the complete waterproofing of the root canal system and that it was biocompatible in contact with the periapical tissues ^1^.

Some endodontic sealers have been proposed as innovative filling materials, however, the ideal root canal sealer has yet to be found because it does not present the main ideal properties that a sealing material requires ^2^. The RCFM must present ideal properties such as the ability for root canal sealing, little or no contraction inside the root canal, antimicrobial activity, insoluble tissue fluids, radiopacity, well tolerated by periapical tissues, and not pigment dental structures ^2^ ^,^ ^3^. One of the basic norms for the choice of endodontic material is associated with the benefits of its physicochemical, biological, and antimicrobial properties ^3^ ^,^ ^4^. A precaution to be taken during the clinical application of these materials for internal use in the pulp cavity is the possibility of changing the color of the dental crown after root canal treatment ^5^ ^,^ ^6^.

Bioactive endodontic materials have been proposed for sealing the root canal system, including calcium silicate-based materials (mineral trioxide aggregate, calcium silicate cement). These materials with similar compositions have been suggested with additional characteristics that allow an improved clinical application, to facilitate handling and manipulation and minimize the coronal discoloration. To achieve this, the new calcium silicate cement (also named bioceramics) forms a colloidal structure after hydration and sequentially develops into a hard structure ^3^ ^,^ ^4^.

The etiology of color change in devitalized teeth must be carefully studied, considering the new materials incorporated into contemporary dentistry. Among the main causes of this chromatic alteration, the presence of restorative materials, bleeding inside the pulp chamber, tissue, or debris decomposition, intracanal dressings, and root canal filling materials located inside the pulp chamber can be highlighted ^5^. Tooth color is directly related to the amount and wavelength of light incident on its surface, reflected or absorbed. Dark objects absorb most of the incident light, resulting in no color. The formation of long molecular chains within the tooth structure is responsible for increasing the light absorption index, resulting in its darkening ^6^. Currently, there is much debate about the need to maintain harmonious smiles and white teeth, making it necessary to prevent possible chromatic changes in the tooth structure. The dentin hybridization technique widely used in indirect restorations may be used to achieve this aim ^7^ ^,^ ^8^.

Dentin hybridization consists of applying a universal adhesive system immediately after any dentin intervention. Dentin hybridization prevents contamination of dentinal tubules, as it prevents dentin exposure to saliva, biofilm, impression materials, and temporary restorations ^7^. The performance of dentin hybridization in teeth with exposure to more than 50% of dentin showed satisfactory results after eleven years. By this time, conventional dentin etching (37% phosphoric acid) and conventional adhesive systems were used. More recently, universal adhesive systems are preferred, especially when dealing with vital teeth ^9^ ^.^ ^10^ ^.^ ^11^ ^.^ ^12^ ^.^ ^13^ ^.^ ^14^ ^.^ ^15^. It has been suggested that the application of a layer of flow composite (resin coating) right after dentin hybridization can eliminate infiltrations and improve the bond strength with resin cement ^15^.

Spectrophotometry (measurement of light absorption or transmission) is one of the widely used techniques for color assessments ^17^ ^,^ ^18^ ^,^ ^19^ ^,^ ^20^ ^,^ ^21^. The spectrophotometers offer accuracy, standardization, and numerical color expression, determined through CIELab system parameters (L*, a*, b*), in which L* indicates luminosity, ranging from 0 (black) to 100 (white), and a* and b* indicate hue (a* represents saturation on the red-green axis and b* on the blue-yellow axis). In studies to assess coronary chromatic changes in dental elements, the L* values are explored with the numerical values of the color variation, as this represents the main coordinate for the analysis of stains (darkening). With this system, any color can be objectively specified with the coordinates L*, a*, and b*^17^ ^,^ ^18^.

Considering the influence on tooth color caused by endodontic sealers and the time between the filling session and the chromatic alteration, it seems reasonable to assess the influence of dentin hybridization with different endodontic sealers and Portland cement on dental color alteration.

## Material and Methods

The 200 healthy bovine central incisors of animals under 3 years of age were immersed in 5% sodium hypochlorite (Fitofarma, Goiânia, Goiás, Brazil) for 30 minutes to remove organic tissue and then placed in 0.2% thymol solution. Digital periapical radiographs of all teeth were taken to verify the following inclusion criteria: absence of calcifications, absence of internal resorptions, and absence of root canal obliteration. The roots of the bovine teeth were measured with a digital pachymeter and sectioned 10mm below the cementoenamel junction with a cooled diamond disc in low rotation (Komet, Besigheim, Germany).

The teeth were initially cleaned with pumice stone and water to prepare the dental substrate, using a rubber at low rotation. Then, access cavities were prepared with 1013 HL spherical diamond burs (KG Sorensen, Cotia, São Paulo, Brazil). Finally, all root canals were prepared with BioRaCe nickel-titanium rotary instruments (FKG Dentaire SA, La Chaux-de-Fonds, Switzerland), following the sequence BR0 25/.08, BR1 15/.05, BR2 25/.04, BR3 25/.06, BR4 35 /.04, and BR5 40/.04, with a speed of 600 rpm and torque of 1.5 Ncm.

Complete debridement of the pulp tissue was performed with the aid of irrigation of 10 mL of 1% sodium hypochlorite, 10 mL of 17% of EDTA (pH 7.2) under agitation for 2 minutes, and final rinse with 10 mL of distilled water. The root canals were dried with absorbent paper cones (Dentsply Sirona, York, Pennsylvania, USA).

The sample was divided into two experimental groups (with and without dentin hybridization), with 38 teeth being distributed to each experimental sealer (19 teeth with dentin hybridization, 19 teeth without dentin hybridization, and 10 teeth for the control group).

Then, dentin hybridization was performed on group 1, applying the Single Bond Universal adhesive system (3M, Minnesota, USA). The application was carried out in the coronal chamber following instructions of the manufacturer: 1- Active application of the adhesive through friction for 20 seconds and air-jet for 30 seconds at 15cm; 2- Light cure for 20 seconds with 1200 mW/cm2 LED (Valo, Ultradent, South Jordan, USA).

The root canal filling was then carried out. The Sealapex (Kerr, Joinville, SC, Brazil) and AH Plus (Dentsply Sirona, York, Pennsylvania, USA) sealers are presented in the form of paste/paste and were mixed in equal quantity until they reached a uniform aspect and ideal consistency and then were taken to the interior of the root canal with gutta-percha cones. The Bio-C Sealer (Angelus, Londrina, PR, Brazil) is ready-to-use and presented in a syringe and was injected directly into the canal with the proper tip. The Endofill (Dentsply Sirona) is presented in powder/liquid form and was mixed in the proportion of one measure of powder to each drop of liquid, in a glass plate, for 10 seconds, until it reached a uniform appearance and ideal consistency. Then it was taken to the interior of the canal with gutta-percha cones. Portland cement (Itaú S/A, Itaú de Minas, MG, Brazil) is presented in powder form and spread on a glass plate by incorporating distilled water until it reaches a homogeneous appearance and ideal consistency.

After the root canal was filled with the endodontic sealers, the coronary chamber was cleaned with a cotton ball soaked in 70% alcohol. A cotton ball was placed in the coronary access, at the level of the cementoenamel junction, to prevent the filling material from invading the coronary chamber. Then the coronary sealing was performed with Coltosol restorative material (Coltene Holding, Altstatten, Switzerland). The color of all specimens was measured, and the sample was stored in an incubator at 100% humidity and 37°C during the experiment.

To verify the influence of dentin hybridization on the final chromatic result of the tooth, the color was measured in both groups with an Easyshade® spectrophotometer (Vita, BadSäckingen, Germany), at four different times: 1- determination of the dental substrate color before filling the root canal; 2- 7 days after root canal filling; 3- 60 days after root canal filling; 4- 180 days after root canal filling.

The analysis (readings) was taken in the same environment, under the same lighting, against a standardized white background (Standard for 45º, 0º Reflectance and Color; Gardner Laboratory Inc., Bethesda, Maryland), by an experienced operator (with more than 10 years of training) who was unaware of which group the samples belonged to, and the spectrophotometer was calibrated at each color measurement of five specimens. The measurement was always carried out in the central area of the tooth crown by making a silicone condensation guide designed to ensure that all samples were evaluated in the same region.

For the determination of crown color, some studies were previously analyzed to establish the parameters to be used ^17^ ^,^ ^18^ ^,^ ^19^ ^,^ ^20^ ^,^ ^21^.

The color was determined through the parameters of the CIELab system (L* a* b*), in which L* indicates the luminosity where the average varies from 0 (black) to 100 (white) and a* and b* the hue, where a* represents saturation on the red-green axis and b* on the blue-yellow axis. Any color can be specified with the L*, a*, and b* coordinates with this system. The coordinates L*, a*, and b* were measured three times in each specimen, and then the values found were averaged to ensure greater accuracy of the measurements. The color variation, represented by ΔE*, was calculated from the formula ^18^:

$$∆E*={\left[{\left(∆L*\right)}^{2}+{\left(∆a*\right)}^{2}+{\left(∆b*\right)}^{2}\right]}^{0.5}$$

where:

∆L* = L1* - L0*;

∆a* = a1* - a0*;

∆b* = b1* - b0*.

Values of ΔE* ≥ 3.3 are considered clinically noticeable.

Data for L* and ΔE* were tabulated and evaluated for normality and homogeneity using the Shapiro-Wilk and Levene tests, respectively. As most of the data were normally and homogeneously distributed, a two-way ANOVA was performed using a two-way ANOVA with Sidak’s post-test for multiple comparisons, with a significance level of 5% (α = 0.05). Analysis was performed using SPSS version 21.0 (IBM Statistics, Armonk, United States).

## Results

For L0 values (Table 1), there was no influence of the sealer factor (p=0.113) or the hybridization factor (p=0.634); however, there was an influence of the interaction between the factors (p=0.018). For the Bio-C Sealer and AH Plus sealers, there was a statistically significant difference between the hybridized and non-hybridized groups (p<0.05). For the other sealers, there was no difference in L0 values regarding hybridization (p>0.05) (Table 1).

**Table 1:** Means and standard deviations (SD) for L* in the 4 evaluation times for each group.

Sealer	Hybridization	L0	L7	L60	L180
	Yes	89.43 (2.01)	89.79 (5.25)	90.55 (3.07)^BC^	90.94 (3.19)^AB^
	No	89.45 (2.97)	91.75 (2.18)	89.69 (4.50)^AB^	92.41 (2.14)^BC^
Bio C-Sealer	Yes	89.14 (2.73)^a^	92.02 (2.01)	93.29 (3.51)^AB^	92.66 (1.56)^B^
	No	90.81 (1.08)^b^	91.72 (3.26)	91.92 (2.61)^B^	94.06 (2.48)^C^
AH-Plus	Yes	90.51 (2.88)^a^	91.37 (2.89)	94.87 (2.57)^aA^	91.70 (1.99)^B^
	No	88.59 (2.60)^b^	90.63 (3.15)	91.02 (3.48)^bAB^	92.59 (2.05)^BC^
Portland	Yes	89.34 (3.25)	91.23 (2.41)	89.75 (2.94)^C^	89.15 (7.10)^AB^
	No	88.84 (2.66)	89.52 (3.13)	88.31 (3.31)^A^	90.03 (2.87)^AB^
Endofill	Yes	90.65 (1.66)	91.55 (3.55)	87.64 (4.11)^C^	87.59 (6.61)^A^
	No	89.12 (1.78)	91.47 (2.99)	88.65 (2.49)^A^	88.21 (2.16)^A^
Control	Yes	90.90 (1.41)	91.34 (2.03)	91.39 (2.19)^ABC^	91.40 (2.37)^A^
	No	92.05 (1.72)	88.17 (2.51)	88.86 (2.36)^AB^	88.67 (2.33)^ABC^

Values followed by different superscript lowercase letters show a statistical difference regarding the performance of hybridization for the same cement (p<0.05). Values followed by different superscript uppercase letters show a statistical difference between the cements within the same hybridization (p<0.05). Values without any superscript did not show any statistical difference (p>0.05).

Considering the L7 values, there was no influence of the sealer factor (p=0.223) or the hybridization factor (p=0.186). There was also no influence of the interaction between the factors (p=0.117). Therefore, no statistical difference exists between any sealer or hybridization in L7 values (p>0.05) (Table 1).

Regarding the L60 values, there was an influence of the sealer factor (p=0.000) and the hybridization factor (p=0.005). However, the effect of the interaction between the factors was not noticed (p=0.058). For the AH Plus sealer, there was a statistically significant difference between the hybridized group and the non-hybridized group in L60 values (p<0.05) (Table 1).

There was also a statistically significant difference for L60 between the Sealapex and AH Plus sealers; AH Plus with Portland and Endofill; and Bio-C Sealer with Portland and EndoFill when hybridized (p<0.05). However, when not hybridized, there was only a statistically significant difference between the Bio-C Sealer with Portland and Endofill (p<0.05) (Table 1).

For the values of L180, there was an influence of the sealer factor (p=0.000), but there was no influence of the hybridization factor (p=0.482) nor of the interaction between the factors (p=0.719). When hybridized, there was a statistically significant difference between Endofill and Control sealer with Bio-C Sealer and AH Plus (p<0.05). In addition, there was a statistically significant difference between the sealers Endofill with AH Plus, Bio-C Sealer, and Sealapex and Portland with Bio-C Sealer when not hybridized (Table 1).

For Group 1, some had an increase in L* (Sealapex, Bio-C Sealer, AH Plus), others decreased (Endofill and Portland cement), and the control group remained more or less stable. For G2, there was an increase in L* for Bio-C Sealer, AH Plus, and Portland and a decrease for Endofill and Control. In addition, Sealapex sealer increased after 7 days, decreased after 60 days, and increased again after 180 days (Table 1).

**Figure 1:**
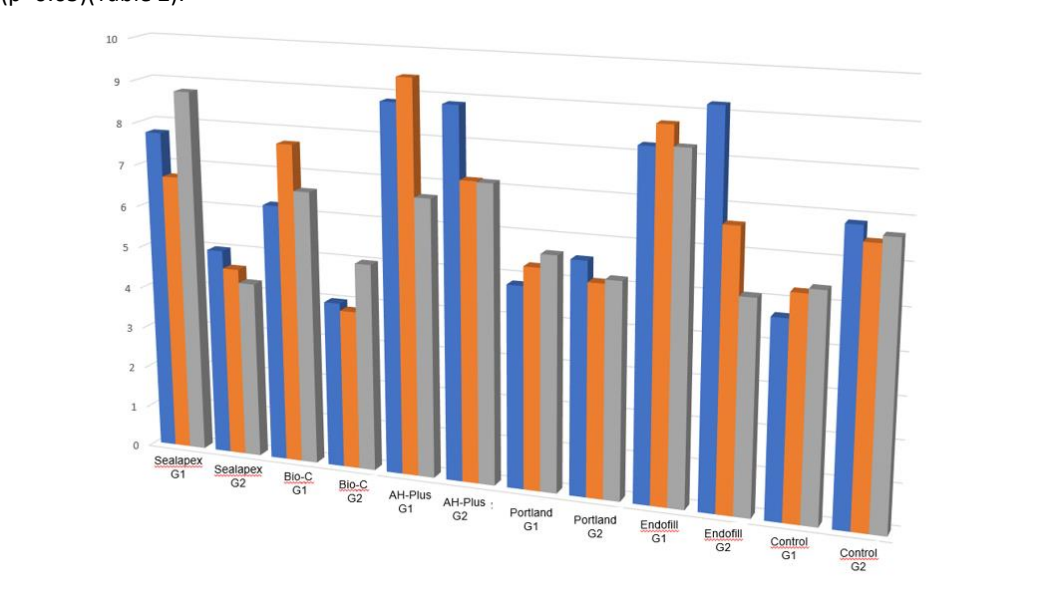
Graphic of the means of ΔE * at each time in the groups with and without hybridization.

Regarding the values of ΔE7 (Figure 1), there was an influence of the sealer factor (p=0.000), but there was no influence of the hybridization factor (p=0.743) nor the interaction between the factors (p=0.085). Considering the statistical difference regarding hybridization performance for the same sealer, there was a statistically significant difference only for the Sealapex sealer (p<0.05). For the other sealers, there was no statistical difference in the values of ΔE7 regarding hybridization (p>0.05)(Table 2).

For the values of ΔE60, there was an influence of the sealer factor (p=0.000) and the hybridization factor (p=0.005), but there was no influence of the interaction between the factors (p=0.175). Only for the sealers Bio-C Sealer and AH Plus, there was a statistically significant difference between the hybridized and non-hybridized groups (p<0.05). For the other sealers, there was no difference in the values of ΔE60 regarding hybridization (p>0.05). Comparing the sealers when hybridized, there was a statistically significant difference for the AH Plus sealer with Portland and control; and for Endofill with Portland and control (p<0.05). When not hybridized, there was only a significant difference between the Bio-C Sealer and AH Plus sealers (p<0.05) (Table 2).

For the values of ΔE180, there was no influence of the sealer factor, the hybridization factor, or the interaction between the factors (p>0.05). Thus, there was no statistically significant difference regarding the performance of hybridization for the same sealer, nor between the sealers within the same hybridization (Table 2).

**Table 2:** Means and standard deviations (SD) for ΔE * in the 3 evaluation times for each group.

Sealer	Hybridization	ΔE7		ΔE60		ΔE180	
		Mean	DP	Mean	DP	Mean	DP
Sealapex	Yes	7.75^aAB^	4.65	6.71^AB^	3.27	8.77	5.38
	No	5.01^bA^	2.82	4.59^AB^	2.76	4.27	3.10
Bio C Sealer	Yes	6.22^AB^	3.16	7.70^aAB^	3.10	6.61	2.80
	No	4.01^A^	2.38	3.84^bA^	2.12	5.01	1.72
AH-Plus	Yes	8.84^B^	3.62	9.42^aB^	3.30	6.69	3.09
	No	8.87^BC^	4.18	7.17^bB^	3.55	7.15	3.94
Portland	Yes	4.85^A^	3.11	5.13^A^	2.24	5.63	5.67
	No	5.57^AB^	3.40	5.08^AB^	3.31	5.18	3.66
Endofill	Yes	8.24^AB^	4.88	8.74^B^	5.24	8.26	6.53
	No	9.22^C^	3.35	6.64^AB^	3.91	5.08	3.74
Control	Yes	4.69^A^	1.66	5.28^A^	2.26	5.39	2.33
	No	6.87^ABC^	4.63	6.51^AB^	4.16	6.67	4.53

Values followed by different superscript lowercase letters show statistical difference regarding the performance of hybridization for the same cement (p<0.05). Values followed by different superscript capital letters show statistical difference between cements within the same hybridization (p<0.05).

## Discussion

The possible change in coronal color after root canal treatment has been a frequent concern during endodontic clinical management. The results of this study showed that all tested endodontic sealers promoted clinically noticeable coronary chromatic alterations during the experimental periods (7 days - 180 days) regardless of dentin hybridization.

Color changes of endodontically treated teeth have been widely studied due to the new materials incorporated into dentistry in recent years ^1^ ^,^ ^2^ ^,^ ^3^ ^,^ ^4^. Root canal filling materials can be highlighted among the causes of this chromatic alteration, including endodontic sealers ^5^. Since the 1990s, dentin hybridization has been studied and emerged as an alternative to prevent contamination of dentinal tubules and, consequently, staining tooth structures ^7^ ^,^ ^11^ ^,^ ^12^ ^,^ ^13^ ^,^ ^14^ ^,^ ^16^.

A concern during the planning of the present study was the reduction of variables, which could interfere with the results of color change after root canal treatment. The choice of bovine teeth was adopted based on analyses of previous studies ^22^ ^,^ ^23^ ^,^ ^24^. The number and the diameter of dentin tubules in root canals of human and bovine teeth were evaluated in the cervical, middle, and apical thirds using scanning electronic microscopy photomicrographs ^22^. The cervical third of the root presented the highest mean values related to the number and diameter of the dentin tubule, followed by the middle and apical thirds, for both specimens studied; the bovine specimens presented a significantly higher mean value, compared with human specimens, about the number of dentin tubules; however, there were no statistically significant differences about the diameter of dentin tubules in the two samples. In the current study, bovine dentin was adopted for greater control between sample variations compared to human teeth (such as size, shape, age control, and healthiness). In this comparative study, the materials, density, and volume of dentinal tubules compared to human dentin should be considered. However, even so, care must be taken not to directly extrapolate the results of laboratory studies to the clinical context.

In the present study, the AH Plus presented the greatest chromatic alterations when hybridized after 7 days, and the Endofill sealer had the greatest chromatic alterations when not hybridized after 7 days. Concerning chromatic alterations after 60 days, the AH Plus obtained the greatest chromatic alterations when hybridized and when not hybridized. Finally, after 180 days, the Sealapex endodontic sealer showed the greatest chromatic alterations when hybridized, and the AH Plus sealer had the greatest chromatic alterations when not hybridized. Thus, it appears that the behavior of endodontic sealers over time against dentin hybridization differs from each other and more than one factor seems to influence the staining of tooth structures, in addition to the different radiopacifiers. Portland cement is a compound of tricalcium and dicalcium silicate without the presence of any radiopacifying elements. It was noted that this material also caused clinically perceptible chromatic changes over time, regardless of dentin hybridization. For the endodontic sealers when compared within the same hybridization process, there was a significant difference of ΔE7 for the AH Plus sealer with Portland and the control when hybridized (p<0.05). When not hybridized, there was a statistically significant difference for the AH Plus sealer with Sealapex and Bio-C Sealer, to Portland with Endofill; and for Endofill with Sealapex and Bio-C Sealer (p<0.05) (Table 2).

The endodontic sealers behaved differently and presented different reactions over time regarding coronary chromatic alteration ^25^ ^,^ ^26^ ^,^ ^27^ ^,^ ^28^ ^,^ ^29^ ^,^ ^30^ ^,^ ^31^. The composition of endodontic sealers showed variations in several factors such as the shape and size of the particles, distribution of these particles, and chemical composition, especially in the different radiopacifiers present in endodontic sealers ^25^. For example, Sealapex uses bismuth oxide as a radiopacifier. Bio-C Sealer uses zirconium oxide with steel oxide and calcium aluminate. AH Plus uses zirconium oxide Endofill uses zinc oxide and bismuth. The dental color alteration and the chemical interaction of bismuth oxide with the main components present in composite (methacrylate) and dentin (collagen) were observed ^26^. The color of WMTA Angelus is affected by contact with dental structures. Collagen, which is present in the organic dentin matrix, reacts with bismuth oxide, resulting in a grayish discoloration. The use of an alternative radiopacifier to replace bismuth oxide in WMTA Angelus is indicated ^26^. Coronary chromatic changes caused by bismuth in contact with different substances were evaluated ^27^ through photographs and color measurements with spectrophotometry. It was found that when in contact with irrigating solutions, especially sodium hypochlorite, bismuth oxide generates severe tooth stains with dark brown-black tones due to the formation of sodium bismuthate. A systematic review of in vitro studies ^29^ determined the effect of different calcium silicate-based cement on dental tissue discoloration. The results clearly showed that some calcium silicate-based cement has a high potential for staining the hard tissues of the tooth. On the other hand, some showed only a small change of color that was not noticeable to the human eye. The effect of endodontic sealer (AH Plus, Endofill, Endométhasone N, Sealer 26) remnants on tooth color was determined ^30^, testing the hypothesis that sealers cause coronal color changes (24 hours and 6 months after RCT). Endodontic sealer remnants affect tooth color confirming the experimental hypothesis. The influence of endodontic filling material was evaluated in a cross-sectional study, as the cervical limit of root filling, and tooth location on the color variation from 1 to 60 months of follow-up ^31^. The following variables were considered in the comparisons: filling material (AH Plus, Endofill, Fillcanal, MTA Fillapex), cervical limit of root filling (dental cervix or ≥2 mm in the apical direction), and tooth location (anterior or posterior teeth) in 83 root canal treatment (RCT). Higher color variation values were yielded from ZOE and MTA-based filling materials, anterior teeth, and cervical limit of root filling not performed at 2 mm below the dental cervix. This fact indicates that endodontic material composition and procedures should be considered to avoid postoperative tooth discoloration, mainly in anterior teeth due to their thinner structure ^31^.

The possibility of changing the color of the crown after RCT has stimulated studies to solve this unpleasant problem. Dentin hybridization can prevent bacterial contamination of dentinal tubules, as it avoids exposure of dentin to saliva, and the diffusion of chemical constituents of endodontic sealer materials, in addition to favoring provisional restorations. The dentin hybridization technique through its use in the inhibition of coronary infiltration by methylene blue after RCT was evaluated by Maruoka et al. ^16^. The group that received dentin hybridization had lower penetration and staining when compared to the group that did not receive dentin hybridization.

Other variables need attention regarding these results for clinical use. First, there is the influence of biological factors in the clinical environment that, despite trying to reproduce them in vitro studies, the time factor is of great relevance in their analysis. Second, other conditions are relevant and may influence the results of colorimetric analysis studies, such as the sample storage medium, correct application of the adhesive, endodontic treatment steps, method selected for colorimetric analysis, and dentin status.

The choice of Coltosol for the present study took into account the results of previous studies, as well as the use of the Single Bond Universal adhesive system. The application of adhesive systems with low load in the performance of dentin hybridization promotes more effectively the maintenance of the integrity of the hybrid layer ^13^.

One limitation of this type of study was the use of bovine central incisors of animals, whose selection was based on an attempt to minimize the variables related to sample selection. However, care must be taken when extrapolating results. A recent systematic review ^32^ searched for evidence to use bovine teeth as substitutes for human teeth in laboratory studies testing several properties. Considering 60 articles evaluated, 15.5% indicated bovine teeth as substitutes for human teeth in laboratory studies, 15.5% indicated substitution with caution, 31% presented the possibility of substitution, 8.7% did not indicate the substitution and 29.3% did not specify the decision. The results showed that in general there is the possibility of human tooth replacement by bovine, both for enamel and dentin substrates, in microleakage studies, organic and inorganic analysis, coefficient of thermal expansion, spectrofluorometry, hardness and microhardness, morphology and radiodensity ^32^.

Few studies in the literature discuss the effectiveness of dentin hybridization on coronary color change caused by endodontic sealers. The development of an ideal endodontic sealer is still sought because, despite advances and technologies applied to contemporary dental materials, there is still no material that combines all the characteristics considered ideal for an endodontic sealer. Future studies with long-term and well-conducted clinical trials are needed to analyze dentin hybridization to prevent coronary chromatic changes in a sample that includes human teeth.

In summary, the results of the present study showed that dentin hybridization influenced the coronary chromatic alteration of the Sealapex subgroup after 7 days and in the Bio-C Sealer and AH Plus subgroups after 60 days. All endodontic sealers caused clinically noticeable coronary chromatic alterations after 7 days, 60 days, and 180 days regardless of dentin hybridization. The studied endodontic sealers behaved in different ways regarding the coronary chromatic alteration, making it difficult to predict a particular behavior.
